# Risk of anterior interosseous nerve injury during forearm surgery: a cadaveric study

**DOI:** 10.1007/s00068-025-02869-9

**Published:** 2025-05-06

**Authors:** Arnaud Walch, Hugo Despert, Clément Jubelin, Laurent Mathieu, Camille Brenac, Thibault Druel

**Affiliations:** 1 Hopital Edouard Herriot, Lyon, France; 2 Hôpital d’instruction des Armées Desgenettes, Lyon, France

**Keywords:** Anterior interosseous nerve, Anatomy, Dissection, Forearm fractures, Cadaveric study

## Abstract

**Purpose:**

The anterior interosseous nerve (AIN) is susceptible to injury during forearm surgery, particularly open reduction and internal fixation (ORIF) of radius fractures. This study aimed to analyze the anatomical relationships between the AIN and the radius to identify regions most vulnerable to iatrogenic injury.

**Methods:**

A cadaveric study was conducted on ten fresh, non-embalmed forearms. Standardized dissections were performed to assess the course of the AIN, its motor branches, and their proximity to bony landmarks. Measurements were taken using a graduated ruler, with reference to the radius, the bi-epicondylar and bi-styloid lines.

**Results:**

The AIN originated, on average, 13 mm from the radius, initially separated from the bone by the flexor digitorum profundus and flexor pollicis longus. The first branch to the flexor pollicis longus emerged at an average of 8 mm from the radius, marking the start of the nerve’s close contact with the bone. The highest risk zone for AIN injury was identified at the junction of the proximal and middle thirds of the forearm.

**Conclusion:**

The AIN and its motor branches exhibit significant anatomical variability but consistently demonstrate proximity to the radius at the proximal-middle third junction. To minimize iatrogenic injury, care should be taken when exposing the anterior radius, particularly by avoiding excessive traction or deep retractor placement in this region.

## Introduction

The anterior interosseous nerve (AIN) arises as a branch of the median nerve. Emerging from its radial aspect, it courses along the anterior surface of the ulnar head of the pronator teres muscle. It then passes between the humeral and ulnar heads of this muscle before passing beneath the fibrous arch of the flexor digitorum superficialis (FDS). Continuing its course adjacent to the interosseous membrane, the AIN plays a crucial role in motor innervation of the anterior forearm compartment. Specifically, it innervates the flexor pollicis longus (FPL), the flexor digitorum profundus (FDP) for the index and middle fingers, and the pronator quadratus muscle [1]. The variability in the number of motor branches has been reported in the literature [2, 3].

The AIN can be injured due to trauma, compression syndromes, or inflammatory diseases, primarily leading to flexion deficits of the thumb’s interphalangeal joint and the index finger’s distal interphalangeal joint, significantly impairing hand dexterity [4]. Several authors have reported neurapraxia of the AIN and its branches in cases of forearm fractures, particularly in diaphyseal fractures of the radius [5–11]. This vulnerability is attributed to the nerve’s close anatomical relationship with the forearm’s bony structures. Potential mechanisms of injury include displacement of bone fragments or excessive traction on the nerve by a surgical retractor [7, 9].

The anatomy of the AIN, its anatomical variations, and potential compression sites have been well documented in the literature [2, 3, 12, 13]. However, its precise relationship with the radius remains unclear. This knowledge gap is significant, as a better understanding of these anatomical relationships is crucial for preventing intraoperative complications, particularly during open reduction and internal fixation (ORIF) of forearm fractures. The aim of this study was to analyze the anatomical relationships between the AIN and the radius and to identify the regions most susceptible to iatrogenic injury during ORIF of forearm fractures.

## Materials and methods

All dissections were performed accordingly to the ethical standards of the research committee of the anatomy laboratory and were in alignment with the Helsinki Declaration of 1964, its subsequent amendments, or comparable ethical benchmarks.

### Protocol

An anatomical study was conducted from January to March 2023 at the Anatomy Laboratory of the Faculty of Medicine, Lyon, France. Dissections were performed on ten fresh, non-frozen, and non-embalmed forearms from whole-body cadavers by two practitioners (TD, CJ). Forearms with surgical scars, callus formation, or apparent bone malunion indicative of prior trauma were excluded from the study.

The study included five female and five male subjects. The mean age at death was 90 years (range, 79–98 years). The average forearm length was 256 mm (range, 243–270 mm).

A standardized protocol was established to investigate the anatomical relationships between the AIN, its branches, and the radius. The cadavers were positioned supine, with the limb abducted and placed on an arm table. Dissections were performed according to the following steps:

- Step 1: “H”-shaped skin incision of the forearm to fully expose the underlying structures.
- Step 2: Identification of the median nerve at the level of the medial bicipital groove.
- Step 3: Dissection of the median nerve from proximal to distal, including the release of the distal portion of the humeral head of the pronator teres and sectioning of the fibrous arch of the FDS.
- Step 4: Exposure of the median nerve, the AIN, and its motor branches up to the pronator quadratus, ensuring preservation of the deep surfaces of the nerves to maintain anatomical integrity.
- Step 5: Identification of key bony landmarks, including the medial and lateral epicondyles, as well as the ulnar and radial styloids.

**Fig. 1:**
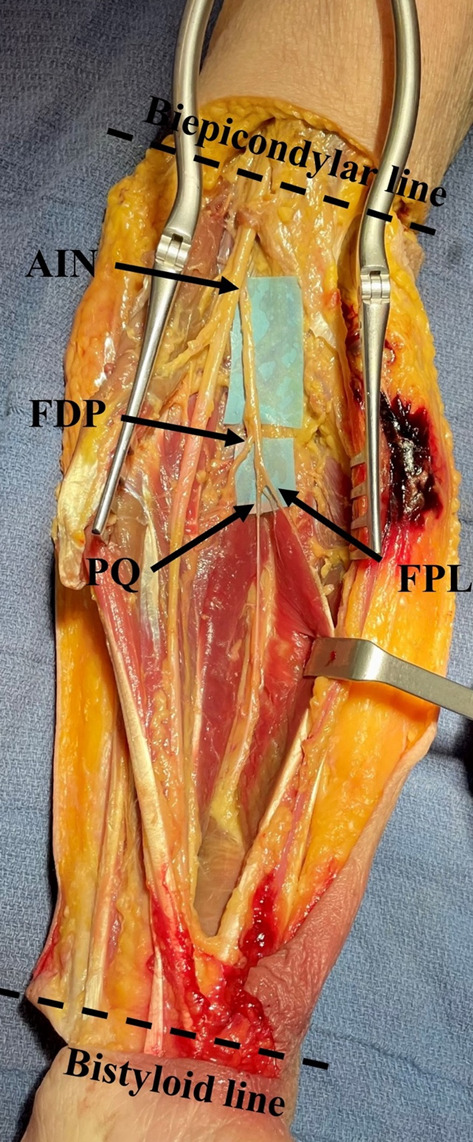
Exposure of the anterior interosseous nerve (AIN) and its branches. Arrows indicate the nerve branches supplying the muscles. AIN, Anterior interosseous nerve; FPL, Flexor pollicis longus, FDP, Flexor digitorum profundus; PQ Pronator quadratus

**Fig. 2:**
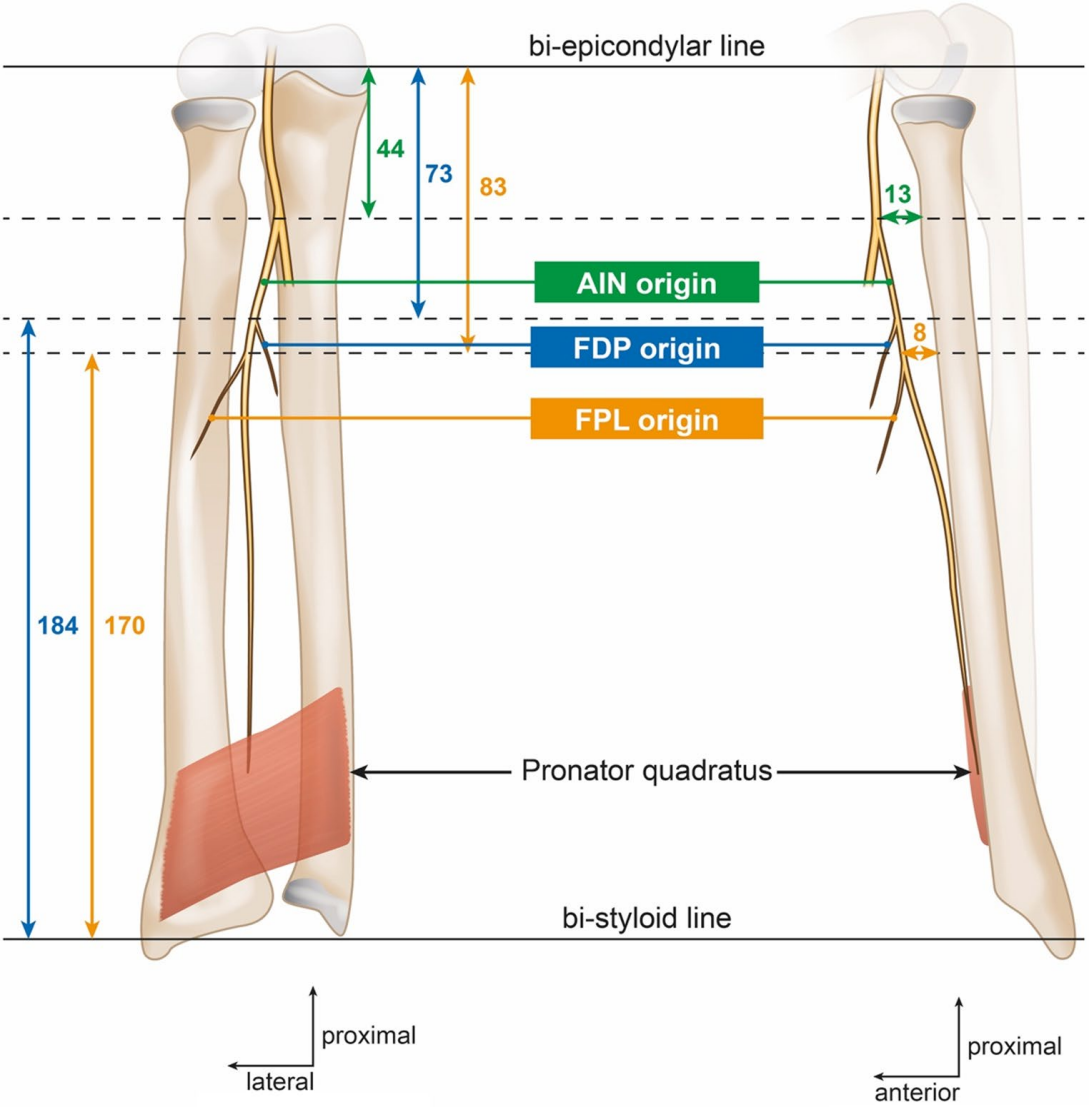
Schematic representation of the anatomical relationship of the anterior interosseous nerve (AIN) and the radius and ulna. FDP: flexor digitorum profundus, FPL: flexor pollicis longue

The anatomy of the different division branches of the AIN was analyzed. Measurements between the AIN and its branches to FPL and FDP, and the radius and bony landmarks, were taken using a graduated stainless steel ruler marked in millimeters, allowing for a measurement precision of ± 0.5 mm, by a single operator (TD). These measurements were recorded with reference to the bi-epicondylar line (proximally) and the bi-styloid line (distally) (Figs. 1 and 2). The distance of these nerves to the radius was assessed at three key points: the origin of the AIN, the origin of the first branch supplying the FPL, and the origin of the first branch supplying the FDP for the index finger.

### Statistics

All continuous variables were reported as mean, standard deviation (SD), and range. To further address the limitation related to sample size, we performed post hoc power analyses on a key anatomical measurement: the distance between the branch to FPL and the radius, assuming a difference of 3 mm would be clinically relevant.

## Results

The origin of the AIN in the forearm was, on average, located 13 ± 3 mm (range, 10–15 mm) from the surface of the radius and was centrally positioned in the forearm (Table 1). At this level, it was consistently separated from the bone by the muscle mass (muscular body) of the FDP and FPL. At the origin of the first branch to the FPL muscle, the AIN was, on average, 8 ± 3 mm (range, 5–10 mm) from the surface of the radius, with its course running immediately along the ulnar border of the radius. After giving rise to the first nerve branch for the FPL, the AIN, in all cases, continued to travel in direct contact with the inter-osseous membrane along the ulnar border of the radius.

In eight cases, the first branch of the AIN was a motor branch to the FPL muscle, while in two cases, it was a motor branch to the FDP muscle of the index finger. In five cases, a motor branch innervated the FDP muscle of the middle finger.

The innervation of the FPL from the AIN was provided by a single branch in 4 cases, two branches in 5 cases, and three branches in 1 case.

**Table 1:** Measurement of the nerve branches in relation to the anatomical bony landmarks

Branches	Radius	Bi-epicondylar line	Bi-styloid line
AIN	13 ± 3 (10–15)	44 ± 10 (25–55)	212 ± 15 (190–235)
Origin of the 1st branch for FDP muscle	Not measured	73 ± 17 (60–83)	184 ± 22 (165–205)
Origin of the 1st branch for FPL muscle	8 ± 3 (5–10)	83 ± 9 (63–93)	170 ± 13 (157–190)

Results are presented in millimeters as mean ± standard deviation (range); AIN, anterior interosseous nerve; FPL, flexor pollicis longus; FDP, flexor digitorum profundus

Based on the measurements obtained from 10 forearms, the standard deviation of the distance between the branch to FPL and the radius was 0.26 mm. Assuming a clinically relevant difference of 3 mm, the calculated effect size (Cohen’s d) was 11.62. With this effect size and sample size, the achieved statistical power was 100% (α = 0.05), indicating that the study was sufficiently powered to detect relevant variations in this anatomical parameter. This result indicates that our sample size was sufficient to detect a small but meaningful anatomical difference, even in the presence of moderate anatomical variability.

## Discussion

The results of this study suggest that the at-risk area for AIN injury is located at the junction of the proximal and middle thirds of the forearm. This risk zone corresponds to the region where the motor branches of the AIN innervating the FPL and FDP muscles are closest to the radius.

The results in the literature are consistent with those reported in this study regarding the measurements of the AIN and the origins of its division branches. The origin of the AIN is typically located 4 to 6 cm from the bi-epicondylar line [2, 3, 12]. In the present study, we observed significant variability in the number of motor branches destined for the FPL and FDP muscles. These findings align with those reported by Ankolekar et al. and Caetano et al., who described 1 to 6 branches for the FDP and 1 to 3 branches for the FPL [2, 12].

These findings support the notion that the AIN and its branches are more vulnerable to injury during fractures at the junction of the proximal and middle thirds of the radius, a theory further supported by several reported cases in the literature. Keogh et al. reported six cases of AIN neurapraxia following ORIF for fractures of the middle third (three cases) or proximal third (three cases) of the radius [8]. Griffiths et al. reported four cases of neurapraxia secondary to ORIF for fractures of the proximal third (three cases) and middle third (one case) of the radius [5]. Additionally, Kajiwara et al. reported a case of AIN neurapraxia with the nerve entrapped at the fracture site in the middle third of the forearm [7]. A few cases of AIN injury have also been reported in fractures of the ulna [5, 7, 9].

Injury to the AIN at the distal third of the forearm appears to have negligible to no functional consequences, as paralysis of the pronator quadratus muscle is typically well compensated by other pronator muscles. However, involvement of the motor branches to the FPL and FDP of the index finger at the proximal/middle third of the forearm has a more significant functional impact. To prevent iatrogenic nerve injury when exposing the anterior aspect of the radius, subperiosteal dissection is crucial to maximize the distance between the nerve and the surgical field. It is preferable to retract the flexor pollicis longus (FPL) muscle from radial to ulnar. We recommend avoiding the use of a double-bent Hohmann retractor along the medial border of the radius, as this may cause neurapraxia due to the close proximity of the nerve branches. Instead, we advise using a Farabeuf retractor—a double-ended handheld instrument—placed superficially to minimize the risk of nerve injury.

In most cases, the AIN provides a branch to the FPL beneath the arch of the FDS muscle. Therefore, the arch of the FDS muscle serves as an important anatomical landmark during an anterior approach to the forearm. Special care should be taken when dissecting around this landmark to avoid injury to the AIN and its branches.

This study is limited by its small sample size and cadaveric nature. However, the results were consistent and provide valuable insights into the anatomical relationships of the AIN with the radius, and our post hoc power analysis confirmed that the sample size of 10 forearms was sufficient to detect a small but meaningful anatomical difference for this distance. The strengths of this study include the reproducibility of the dissection and the precise, sequential analysis of the different nerve branches. Although the average age of the dissected cases is relatively high, advanced age does not appear to be a limiting factor for nerve anatomy analysis in the absence of traumatic or surgical history.

## Conclusion

The present study highlights the close anatomical relationships of the AIN and its motor branches with the radius. Careful attention is required during forearm approaches, especially at the junction of the proximal and middle thirds of the forearm, to prevent iatrogenic nerve injury.

## Data Availability

No datasets were generated or analysed during the current study.
